# Melatonin improves experimental colitis with sleep deprivation

**DOI:** 10.3892/ijmm.2015.2080

**Published:** 2015-01-27

**Authors:** YOUNG-SOOK PARK, SOOK-HEE CHUNG, SEONG-KYU LEE, JA-HYUN KIM, JUN-BONG KIM, TAE-KYUN KIM, DONG-SHIN KIM, HAING-WOON BAIK

**Affiliations:** 1 Departments of Gastroenterology, Eulji University, Daejeon 301-746, Republic of Korea; 2 Biochemistry and Molecular Biology, School of Medicine, Eulji University, Daejeon 301-746, Republic of Korea; 3 Department of Gastroenterology, Ajou University School of Medicine, Suwon 443-721, Republic of Korea

**Keywords:** melatonin, sleep deprivation, colitis

## Abstract

Sleep deprivation (SD) is an epidemic phenomenon in modern countries, and its harmful effects are well known. SD acts as an aggravating factor in inflammatory bowel disease. Melatonin is a sleep-related neurohormone, also known to have antioxidant and anti-inflammatory effects in the gastrointestinal tract; however, the effects of melatonin on colitis have been poorly characterized. Thus, in this study, we assessed the measurable effects of SD on experimental colitis and the protective effects of melatonin. For this purpose, male imprinting control region (ICR) mice (n=24) were used; the mice were divided into 4 experimental groups as follows: the control, colitis, colitis with SD and colitis with SD and melatonin groups. Colitis was induced by the administration of 5% dextran sulfate sodium (DSS) in the drinking water for 6 days. The mice were sleep-deprived for 3 days. Changes in body weight, histological analyses of colon tissues and the expression levels of pro-inflammatory cytokines and genes were evaluated. SD aggravated inflammation and these effects were reversed by melatonin in the mice with colitis. In addition, weight loss in the mice with colitis with SD was significantly reduced by the injection of melatonin. Treatment with melatonin led to high survival rates in the mice, in spite of colitis with SD. The levels of pro-inflammatory cytokines, such as interleukin (IL)-1β, IL-6, IL-17, interferon-γ and tumor necrosis factor-α, in the serum of mice were significantly increased by SD and reduced by melatonin treatment. The melatonin-treated group showed a histological improvement of inflammation. Upon gene analysis, the expression of the inflammatory genes, protein kinase Cζ (*PKCζ*) and calmodulin 3 (*CALM3*), was increased by SD, and the levels decreased following treatment with melatonin. The expression levels of the apoptosis-related inducible nitric oxide synthase (*iNOS*) and wingless-type MMTV integration site family, member 5A (*Wnt5a*) genes was decreased by SD, but increased following treatment with melatonin. Treatment with melatonin reduced weight loss and prolonged survival in mice with colitis with SD. Melatonin exerted systemic anti-inflammatory effects. Gene analysis revealed a possible mechanism of action of melatonin in inflammation and sleep disturbance. Thus, melatonin may be clinically applicable for patients with inflammatory bowel disease, particulary those suffering from sleep disturbances.

## Introduction

Inflammatory bowel disease (IBD), including ulcerative colitis (UC) and Crohn’s disease (CD), are chronic recurrent inflammatory disorders of the gastrointestinal tract characterized by abdominal pain, chronic diarrhea and weight loss. IBD is a chronic debilitating disease with a considerable decline in the quality of life, and the symptoms of IBD occur repeatedly with increasing severity and aggravation (1).

IBD is affected by genetic and environmental factors (2,3); however, their etiology remains unclear. Known environmental factors for IBD, such as smoking, exacerbate the diseases in individuals with genetic susceptibility (4). Chronic stress is a well-known factor that increases the progression and recurrence of IBD, and decreased immunity, caused by stress, may also affect individuals who are more susceptible to IBD (5). Sleep disturbance, a physiological stressor, has been clinically shown to aggravate IBD in patients (6). It has been reported that night shift workers with irregular sleep patterns suffer from IBD more commonly than daytime workers (7). Thus, sleep disturbance affects symptoms in patients with IBD and can aggravate intestinal inflammation. In a previous study, even patients with inactive IBD had more sleep disturbances, prolonged sleep latency, sleep fragmentation, were prone to use a greater amount of sleeping pills, had decreased activity during the day, increased fatigue and lower sleep quality compard with the healthy control group (8).

Melatonin has been shown to relieve inflammation in sleep-related apoptosis and regulates signal transduction (9). Melatonin acts as an antioxidant and an anti-inflammatory agent in lung inflammation with sleep deprivation (SD) (10). The melatonin concentration in the GI tract is 100-fold greater than that in the blood and 400-fold greater than that in the pineal gland (11). Melatonin has been shown to exert anti-inflammatory effects in experimental models of colitis (12–14). However, the role of melatonin in the GI tract, and its connection with SD in particular, remains unclear.

In the present study, we sought to evaluate the effects of melatonin under the specific conditions of colonic inflammation with SD. Thus, to establish the concept that SD aggravates colitis and to determine the effects of melatonin, we used a specially designed model of SD.

## Materials and methods

### Animals

**Figure 1:**
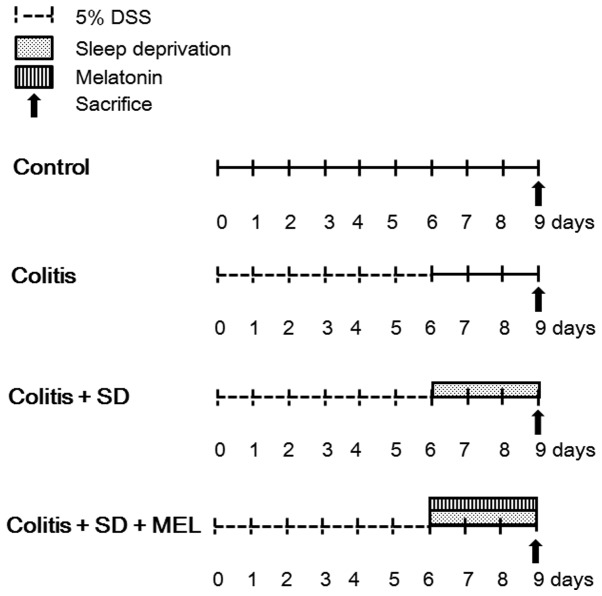
Schematic dagriam of the experiment design. Group I: control (n=6). Group II (n=6): experimental colitis was induced in the mice by the addition of 5% dextran sulfate sodium (DSS) solution in their drinking water overa period of 6 days. Group III: colitis + SD (n=6): mice with DSS-induced colitis were deprived of sleep for 3 days, using the modified multiple platform method. Group IV (n=6): melatonin was administered to the mice intraperitoneally at a dose of 10 mg/kg for 3 days after the induction of colitis with 5% DSS. SD, sleep deprivation; MEL, melatonin.

In total, 24 male imprinting control region (ICR) mice were used (obtained from Daehan Biolink Co. Ltd., Chungbuk, Korea). The mice were 7–8 weeks old and weighed 35–40 g. They were all housed in a modified multiple platform water bath and kept under controlled conditions of temperature (22–24°C), humidity (55–60%) and a 12/12-h light/dark cycle. They were allowed free access to food and water throughout the experimental period. Clinical evaluations, including body weight and stool consistency were made daily. The mice were divided into 4 groups as follows: the untreated control group, the group with dextran sulfate sodium (DSS)-induced colitis, the group with DSS-induced colitis with SD, and the group with DSS-induced colitis with SD administered an injection of melatonin (10 mg/kg) (Fig. 1). We also examined the survival rate of the mice during the whole experimental period. This study protocol was approved by the Eulji University Institutional Animal Care and Usage Committee, Daejeon, Korea (approval no. EUIACUC-09-06).

### Induction of colitis

Experimental colitis was induced by the addition 5% (w/v) DSS (Sigma-Aldrich, St. Louis, MO, USA) to the drinking water for 6 days, from day 0 to 6.

### SD

The mice were subjected to partial SD using a modified multiple platform water bath, as previously described (15,16). A total of 18 platforms were placed in the water tank and 6 mice were placed in the water bath. By jumping, each mouse in the water bath could move from one platform to another. The water bath was filled with water 4 cm from the base. When the mice reached the REM stage of sleep, which is the paradoxical phase of sleep, muscle atonia caused the mice to fall into the water. Subsequently, the awakened mice tried to climb onto a platform in order to avoid drowning. Throughout the experimental period, the water was changed with clean water in the tank. The mice were sacrificed after being subjected to 3 days of SD.

### Administration of melatonin

Melatonin was dissolved in ethanol and then diluted in phosphate-buffered saline (the final concentration of ethanol was 1%). Melatonin was administered to the mice intraperitoneally at a dose of 10 mg/kg for 3 days after the induction of colitis with 5% DSS. Normal saline was administered to the control mice intraperitoneally. The mice were sacrificed after the final administration of melatonin on day 3.

### Tissue collection

On the final day of the experimental period, the mice were sacrificed following treatment with inhalation anesthetics, Enflurane (Minrad Inc., Buffalo, NY, USA). Blood obtained from each animal was collected in sterile tubes and centrifuged (890 × g, 15 min, 4°C). The serum was frozen at −80°C until the cytokine analysis was performed. Colon tissues were obtained from each animal following sacrifice and divided into 3 segments. One segment was used for histopathological examination. The other tissues were snap-frozen in liquid nitrogen for microarray analysis and reverse transcription-quantitative polymerase chain reaction (RT-qPCR).

### Histopathological evaluation

A segment of the colon was fixed in 10% neutral-buffered formalin before embedding in paraffin wax. The cections were cut at 5 *μ*m and stained with hematoxylin and eosin. The sections, placed on poly-lysine-coated slides, were observed under a light microscope (Olympus BX51; Olympus Corp., Tokyo, Japan).

### Analysis of cytokines

Serum from the experimental mice was used to assess the levels of inflammatory cytokines, such as interleukin (IL)-1β, IL-6, IL-17, interferon-γ (INF-γ) and tumor necrosis factor-α (TNF-α) using the Bio-Plex Pro Mouse Cytokine assay kit (Bio-Rad, Hercules, CA, USA).

### Microarray analysis and RT-qPCR

Total RNA was isolated from the mouse colon samples using TRIzol reagent (Invitrogen, Carlsbad, CA, USA). ImProm-II reverse transcriptase (Promega Corp., Madison, WI, USA) and an oligo(dT) primer were used for the synthesis of the cDNA. qPCR was performed with a 20-*μ*l reaction mixture containing 10 *μ*l of SYBR-Green qPCR Premix (Finnzymes Oy, Espoo, Finland), 10 *μ*mol of forward primer, 10 *μ*mol of reverse primer and 1 *μ*g of cDNA using the DNA Engine Opticon System (MJ Research Inc., Waltham, MA, USA). Initial denaturation was performed at 95°C for 5 min. Subsequenlty, 40 cycles of PCR were performed, consisting of denaturation at 95°C for 1 min, annealing at 50–59°C for 30 sec and extension at 72°C for 1 min. The value of threshold cycle (Ct) normalized to the mRNA value of β-actin was used to assess mRNA expression.

### Statistical analysis

Data were initially analyzed by ANOVA. Significant main effects determined by ANOVA were followed by the Tukey-Kramer post hoc test to identify which experimental groups differed significantly from their respective controls. A p-value <0.05 was considered to indicate a statistically significant difference.

## Results

### Weight change and survival rate

Experimental colitis in the ICR mice was induced by the addition of 5% DSS to their drinking water for 6 days. In total 40% of the mice had diarrhea on the third day of treatment with 5% DSS. All the mice showed gross rectal bleeding on the fourth day of treatment with 5% DSS. Bloody diarrhea and gross rectal bleeding in the mice treated with 5% DSS suggested the development of inflammation.

**Figure 2:**
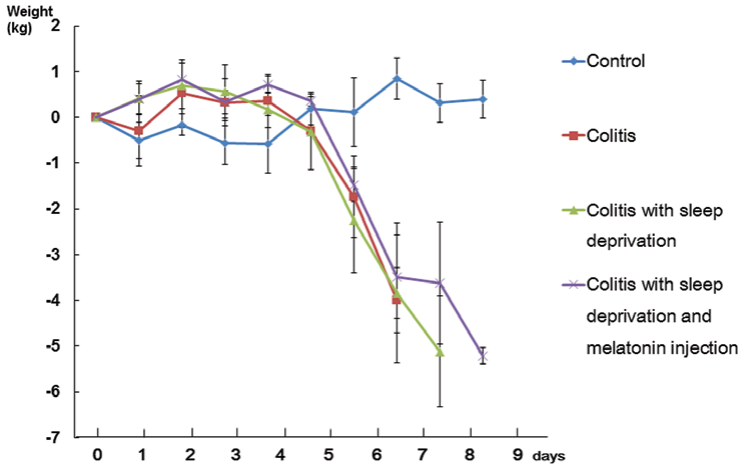
Changes in body weight in each group. The body weight in the group with 5% dextran sulfate sodium (DSS)-induced colitis decreased much more than the control group. The colitis with sleep deprivation group showed severe weight loss, but melatonin treatment significantly reduced weight loss. The weights of individual mice were measured daily. Data represent the means ± SE.

The body weights of the mice in the experimental group are shown as Fig. 2. Weight loss was distinct on the sixth day of the administration of 5% DSS. The colitis with SD group showed severe weight loss; however, treatment with melatonin significantly reduced weight loss. SD in the mice with 5% DSS-induced colitis caused more severe gross rectal bleeding. Treatment of the mice subjected to SD with melatonin did not diminish the gross rectal bleeding compared with the colitis with SD group (data not shown).

**Figure 3:**
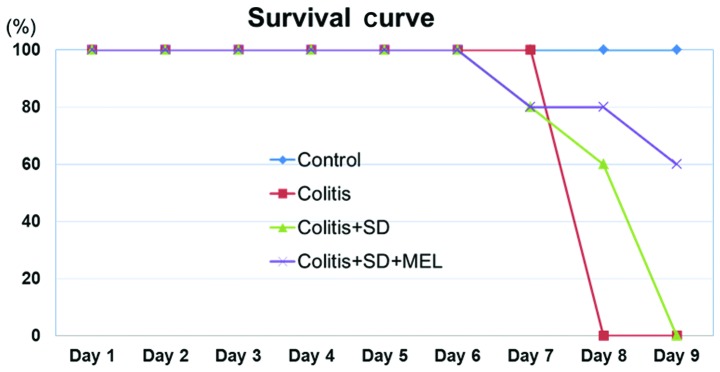
Survival rate of the experimental groups. The group with 5% dextran sulfate sodium (DSS)-induced colitis with sleep deprivation (SD) showed poor survival rates. The melatonin-treated group showed significantly improved survival. MEL, melatonin.

All mice in the group administered 5% DSS died on the third day after 6 days of treatment with 5% DSS. In total, 20% of the mice died on the first and second day of SD and 60% of the mice died on the third day. With melatonin treatment, 20% of the mice died after the first and third day of SD (Fig. 3). In conclusion, the colitis with SD group showed severe weight loss and poor survival during the experiment. However, the melatonin-treated group showed reduced weight loss and prolonged survival.

### Histopathological evaluation

**Figure 4:**
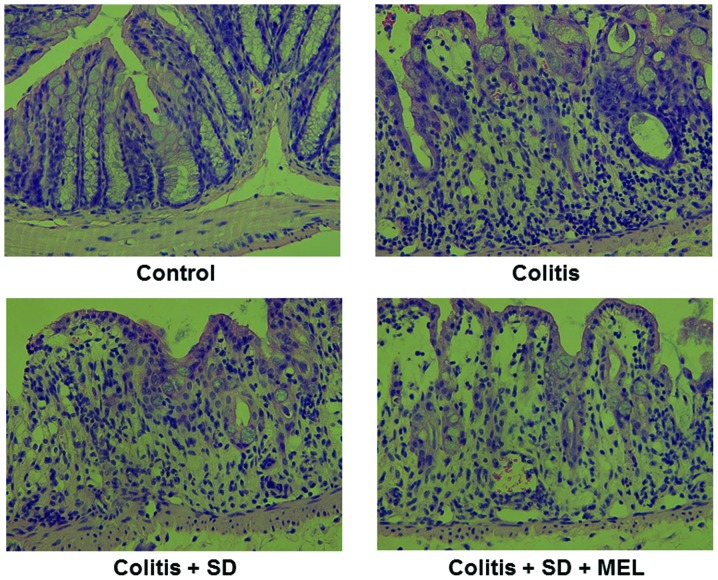
Histological analyses of inflammatory changes due to sleep deprivation and melatonin in 5% dextran sulfate sodium (DSS)-induced colitis (H&E staining, x400). (A) No significant number of inflammatory cells was observed in the colon of the control group. (B) There was erosion with moderately inflamed mucosa in the colons of mice in the group with 5% DSS-induced colitis. (C) Sleep deprivation aggravated the severity of colitis in the mice with 5% DSS-induced colitis. There was erosion with a moderately severely inflamed mucosa in the colons of mice with colitis with sleep deprivation (SD). (D) Melatonin treatment reduced the inflammation in the colon tissue. MEL, melatonin.

In the histological analysis, the colon of the mice with 5% DSS-induced colitis showed edema and infiltration of inflammatory cells into the mucosa (Fig. 4B) compared with the control group (Fig. 4A). SD aggravated the severity of colitis in the group with 5% DSS-induced colitis. Increased numbers of infiltrating cells and mucosal injury were observed with SD. There was erosion with a moderately severely inflamed mucosa in the colon of the mice with colitis and SD (Fig. 4C). Treatment with melatonin led to a decrease in inflammation in the colon tissue. There was reduced erosion with moderately severely inflamed mucosa (Fig. 4D).

### Melatonin and SD: effects on inflammatory cytokines

**Figure 5:**
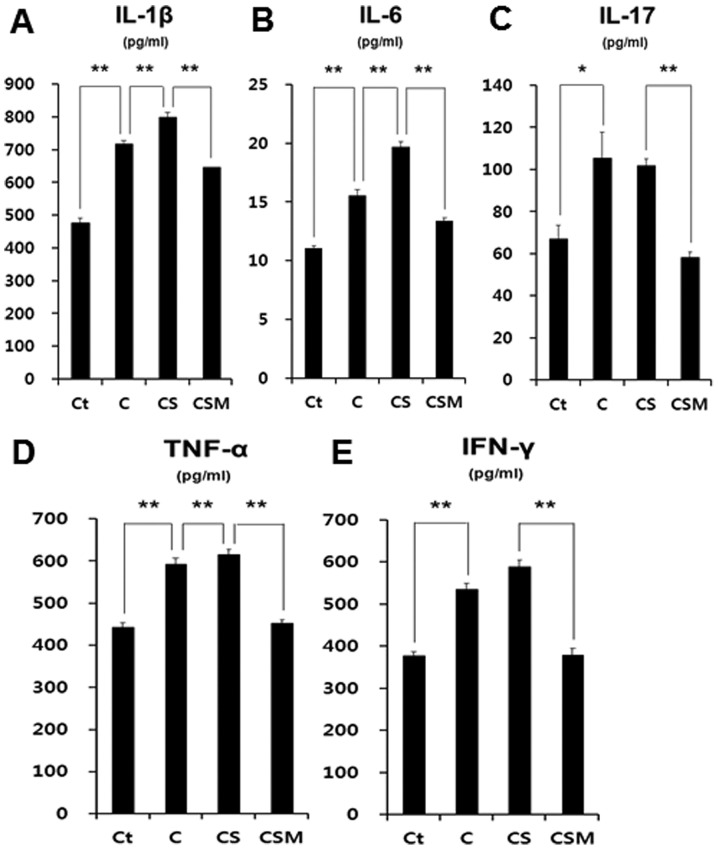
Analysis of pro-inflammatory cytokines in the mice with dextran sulfate sodium (DSS)-induced colitis. (A) Interleukin (IL)-1β, (B) IL-6, (C) IL-17, (D) tumor necrosis factor-α (TNF-α), (E) interferon-γ (IFN-γ). Serum levels of cytokines were measured using a Bio-Plex Pro Mouse Cytokine assay kit. Ct, control; C, colitis; CS, colitis with sleep deprivation; CSM, colitis + SD + melatonin treatment. ^*^P<0.05 and ^**^P<0.01.

There was a significant increase in the levels of IL-1β, IL-6, IL-17, TNF-α and INF-γ in the plasma of the mice with colitis. SD aggravated the increase in the levels of these pro-inflammatory cytokines. However, treatment with melatonin significantly reduced the levels of these cytokines (Fig. 5), demonstrating the systemic anti-inflammatory properties of melatonin.

### Analysis of gene expression by microarray and RT-qPCR

Using microarray analysis and RT-qPCR assays, it was found that SD increased the mRNA levels of protein kinase Cζ (*PKCζ*) and calmodulin 3 (*CALM3*) and decreased those of the inducible nitric oxide synthase (*iNOS*) and wingless-type MMTV integration site family, member 5A (*Wnt5a*) genes significantly. Melatonin increased the mRNA levels of *iNOS* and *Wnt5a1*, and decreased those of *PKCζ* and *CALM3*.

**Figure 6:**
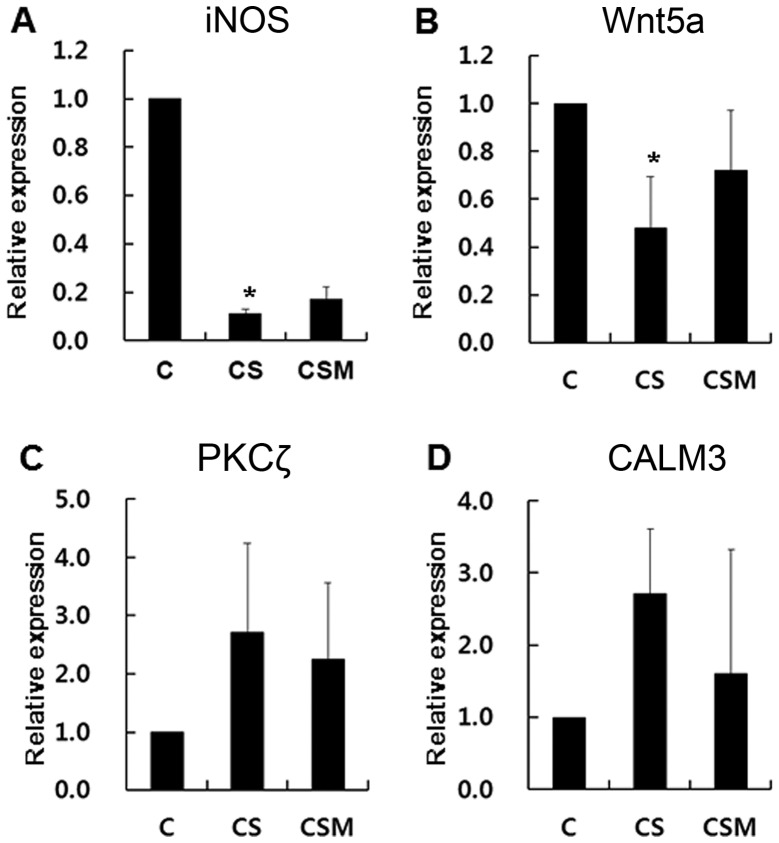
RT-qPCR for validation of microarray data. Sleep deprivation significantly decreased the mRNA levels of (A) inducible nitric oxide synthase (*iNOS*) and (B) wingless-type MMTV integration site family, member 5A (*Wnt5a*), which showed an increasing trend with melatonin treatment. Sleep deprivation increased the mRNA levels of (C) protein kinase Cζ (*PKCζ*) and (D) calmodulin 3 (*CALM3*), which were decreased after the melatonin injection. However, the differences were not statistically significant. ^*^P<0.05.

SD and melatonin simultaneously affected some of the genes. Thus, *iNOS* and *Wnt5a* gene expression in the mice with colitis with SD decreased significantly compared with the colitis group, and their expression levels increased in the mice with colitis with SD treated with melatonin. *CALM3* and *PKCζ* gene expression in the colitis with SD group increased significantly compared with the colitis group, and their expression levels decreased in the melatonin-treated group (Fig. 6).

## Discussion

Patients with IBD frequently identify a significant stressor prior to disease flare-ups, and sleep loss or sleep disturbance is considered one form of physiological stress (17). It was previously reported that SD worsened inflammation and delayed recovery in a mouse model of colitis (18). Thus, SD may be a significant factor aggravating disease activity and flare-ups. In clinical data, it has been reported that patients with IBD had significantly prolonged sleep latency, frequent sleep fragmentation, were prone to use a greater amount of sleeping pills, had decreased daytime energy and increased tiredness (19). A trial to control stress or sleep disturbances in patients with IBD was not effective with psychotherapy alone, as psychotherapy did not improve the disease course or reduce relapse. However, it did improve the disease-specific quality of life (IBDQ) in patients with UC (20).

Recently, our understanding of the circadian rhythm has advanced. The rhythm of the sleep-wake cycle in humans is tightly controlled by the circadian system, which fluctuates with the light-dark cycle. The central circadian clock is located in the hypothalamic suprachiasmatic nucleus (SCN), which sends nerve impulses to the pineal gland and regulates the production of neurohormones, including melatonin (19).

Melatonin has multiple neurohormonal roles. It was discovered in 1958 by Lerner *et al* (21), and is secreted at night time from the pineal gland. It is a derivative of serotonin and inhibits the increase in gastrointestinal motility and smooth muscle cell contraction caused by serotonin (22). It is surprising that melatonin is present in the GI tract at levels 400-fold higher than those in the pineal gland (23). Melatonin is synthesized in enterochromaffin cells throughout the gut and L-tryptophan is a crucial precursor in gut melatonin synthesis (24).

Melatonin has been reported to reduce the severity of experimental colitis in mice and rats, although its mechanisms of action remain unclear. In experimental colitis in rats, melatonin has been shown to reduce colon injury by influencing numerous events, including the enzymatic activities of matrix metalloproteinase (MMP)-9 and caspase-3, by suppressing the activities of cyclooxygenase (COX)-2 and iNOS, inhibiting the expression of nuclear factor-κB (NF-κB), and acting as a radical scavenger (25,26).

In rats with 2,4,6-trinitrobenzenesulfonic acid (TNBS)-induced colitis, melatonin has been shown to decrease caspase-3 activity, malondialdehyde (MDA) levels, myeloperoxidase activity and NF-κB epxression, and to increase glutathione levels (6). In another study, melatonin was shown to exert anti-inflammatory and anti-apoptotic effects by decreasing TNF-α, IL-1β and Fas ligand expression, the phosphorylation of c-Jun, apoptosis and Bax expression induced by treatment with dinitrobenzene sulfonic acid (DNBS) (7). Moreover, the regulation of macrophage activity (27) and the reduction of bacterial translocation in TNBS-induced colitis has been reported (10).

The results of this study demonstrated that SD increased the severity of inflammation, whereas treatment with melatonin reduced inflammation during SD, histopathologically and clinically. The modified multiple platform method was developed independently as a method of SD (16). Tang *et al* (18) used a rotating wheel to induce SD in a mouse model of colitis. For our experiments, we used a specially designed SD cage. This cage has multiple platforms in a water bath. Previous studies have indicated that SD plays an important role in aggravating IBD (28,29). However, our results are unique in investigating the dual effects of melatonin on both SD and colitis.

The present study demonstrated that mice with colitis with SD showed more severe weight loss than the mice with colitis alone. These results are consistent with those of a previous study demonstrating chronic total SD in rats (30). Ttreatment with melatonin reduced weight loss and prolonged survival under the same stressful conditions of a water bath, SD and colitis. Thus, melatonin not only controls colon inflammation, but also has systemic effects on survival. These effects are likely and at least partly related to the changes in cytokine levels due to SD and melatonin. We found a significant increase in the levels of IL-1β, IL-6, IL-17, TNF-α and IFN-γ in the plasma of mice with colitis. SD aggravated the increase in the levels of pro-inflammatory cytokines; however, treatment with melatonin significantly reduced the levels of these cytokines, indicating systemic anti-inflammatory properties.

This finding is similar to that of a recent study which demonstrated that UC resulted in a significant increase in the plasma levels of pro-inflammatory markers (IL-1β, IL-6 and TNF-α) compared with the control group (31). Furthermore, UC resulted in an increase in systemic genotoxicity, which may be due to the elevated plasma TNF-α levels, as it has been reported that TNF-α and TNF-receptor signaling are of major importance in inflammation-associated systemic genotoxicity in mice (32). Moreover, bacterial translocation to the systemic circulation due to increased gut permeability in mice with UC may be partially involved in the induction of systemic DNA damage, as some gut bacteria have genotoxic genes which are activated by inflammation (33). The mucosal healing capability of melatonin has also been demonstrated, resulting in the maintenance of gut barrier integrity, as indicated by the expression of occludin in the colons of mice with UC. This was associated with reduced plasma lipopolysaccharide (LPS) levels, indicating a decrease in the bacterial translocation to the systemic circulation and the ultimately reduced systemic inflammation and DNA damage with melatonin intervention (31).

In this study, using microarray and RT-qPCR assays of the mouse colon, the mRNA levels of *PKCζ* and *CALM3* were increased by SD, but decreased following treatment with melatonin. However, these effects were not statistically significant in our experiments. PKCζ is a isoform of protein kinase C. It is involved in cell proliferation and cell death (34). Calmodulin is a calcium-binding protein. It plays important roles in metabolism, apoptosis, inflammation and the immune response (35).

Furthermore, the mRNA levels of *iNOS* and *Wnt5a* were decreased by SD, but increased following treatment with melatonin. In previous studies, the expression of *iNOS* in IBD was inconsistent (36,37). Kankuri *et al* (36) reported that the expression of *iNOS* was increased in the colonic mucosa of patients with IBD. In another study, the mRNA level of *iNOS* in the inflamed mucosa was increased in only 1 out of 6 patients. However, 3 out of 6 patients showed a reduced *iNOS* expression in the inflamed mucosa of the colon compared with the uninflamed mucosa (37). This result was in accordance with that of a previous study showing that iNOS was positively regulated by Wnt β-catenin signaling (38). Wnt is involved in several pathways, including cell differentiation, development and degeneration. Toll-like receptor-mediated *Wnt5A* expression plays an important role in macrophage activation (39). SD and melatonin may affect metabolism, gene expression, cell growth and apoptosis in the process of aggravating and relieving IBD.

To date, aminosalicylates, corticosteroids, immunosuppressants and biological agents have been used to control IBD (40). There are many novel candidates, including prebiotics, probiotics, peroxisome proliferator-activated receptor γ agents and new biologics (41). Previous studies have revealed that melatonin exerts antioxidant, anti-inflammatory and anti-apoptotic effects (6–8). Thus, melatonin is considered to have potential as a novel therapeutic agent for IBD. It has already been reported to protect against UC in clinical settings (42,43). In a previous study, the effects of melatonin were evaluated on the activity of the inflammatory process and sustaining remission in patients with UC (42). UC in patients treated with mesalazine with melatonin 5 mg daily at bedtime remained in remission during 12 months of observation with significantly lower C-reactive protein (CRP) levels compared to the control group. The authors concluded that adjuvant therapy with melatonin may help in sustaining remission in patients with UC (42). Melatonin has also been shown to be beneficial in chronic intestinal inflammation and colon cancer, modulating autophagy and sirtuin activity (44).

This study had some limitations. First, we only investigated acute changes in the inflammatory response, such as body weight and bloody diarrhea, and histological changes with SD and melatonin treatment. To examine inflammatory changes due to SD and melatonin completely, changes occurring with longer SD and melatonin treatment periods should be investigated. Second, we only conducted DNA microarray analysis of the colon tissue between the colitis with SD group and the SD with melatonin group. A simple melatonin treatment group should also be evaluated to determine any direct effect of melatonin on inflammation.

In conclusion, in this study, we demonstrated that SD significantly aggravated inflammation and worsened the survival rate, whereas treatment with melatonin decreased inflammation and prolonged survival in mice with DSS-induced colitis. In addition, melatonin decreased systemic inflammatory cytokine production and reversed the changes which occurred due to SD. These results suggest that melatonin may be useful as an adjuvant therapeutic in patients with IBD, particularly in those suffering from sleep disturbances. However, a large randomized controlled study of the effects of melatonin in patients with IBD is required.
